# Annexin A1 as a Regulator of Immune Response in Cancer

**DOI:** 10.3390/cells10092245

**Published:** 2021-08-30

**Authors:** Thaise Gonçalves Araújo, Sara Teixeira Soares Mota, Helen Soares Valença Ferreira, Matheus Alves Ribeiro, Luiz Ricardo Goulart, Lara Vecchi

**Affiliations:** 1 Laboratory of Genetics and Biotechnology, Federal University of Uberlandia, Patos de Minas 387400-128, MG, Brazil; tgaraujo@ufu.br (T.G.A.); saratsm.s@hotmail.com (S.T.S.M.); helensvalenca@gmail.com (H.S.V.F.); matheusribeiromarketing@gmail.com (M.A.R.); 2 Laboratory of Nanobiotechnology, Federal University of Uberlandia, Uberlandia 38400-902, MG, Brazil; lrgoulart@ufu.br

**Keywords:** Annexin A1, tumor microenvironment, immune-suppression, cancer aggressiveness

## Abstract

Annexin A1 is a 37 kDa phospholipid-binding protein that is expressed in many tissues and cell types, including leukocytes, lymphocytes and epithelial cells. Although Annexin A1 has been extensively studied for its anti-inflammatory activity, it has been shown that, in the cancer context, its activity switches from anti-inflammatory to pro-inflammatory. Remarkably, Annexin A1 shows pro-invasive and pro-tumoral properties in several cancers either by eliciting autocrine signaling in cancer cells or by inducing a favorable tumor microenvironment. Indeed, the signaling of the *N*-terminal peptide of AnxA1 has been described to promote the switching of macrophages to the pro-tumoral M2 phenotype. Moreover, AnxA1 has been described to prevent the induction of antigen-specific cytotoxic T cell response and to play an essential role in the induction of regulatory T lymphocytes. In this way, Annexin A1 inhibits the anti-tumor immunity and supports the formation of an immunosuppressed tumor microenvironment that promotes tumor growth and metastasis. For these reasons, in this review we aim to describe the role of Annexin A1 in the establishment of the tumor microenvironment, focusing on the immunosuppressive and immunomodulatory activities of Annexin A1 and on its interaction with the epidermal growth factor receptor.

## 1. Introduction

Neoplastic transformation and progression encompass multiple unknown or poorly described events. In this context, Hanahan and Weinberg proposed different hallmarks that, despite being interrelated, define the principles of tumorigenesis. These hallmarks include the resistance to cell death mechanisms, uncontrolled proliferation, induction of angiogenesis, invasion potential, metastasis formation and immune escape [1]. Cancer cells acquire these hallmarks by modifying the extracellular matrix (ECM) and modulating the behavior of different cell types that surround the tumor. In this way, cancer cells create a unique environment, called the tumor microenvironment (TME), that strongly favors malignancy and cancer dissemination [2]. Unfortunately, the appearance of metastasis profoundly worsens the prognoses of patients, since no efficient therapies are available [3]. It is therefore essential to study the factors that are involved in the establishment of the TME in order to design new therapeutic strategies that could help to avoid cancer spreading and manage metastatic cancer patients. Of particular interest is the understanding of how cancer cells create a TME that favors immune evasion. In this context, it is important to unravel the immunomodulatory role of the protein Annexin A1 (AnxA1), which is the purpose of this review.

## 2. Anticancer Immune Response and Cancer Immune Escape

The immune system recognizes antigens expressed on the surface of transformed cells, leading to their destruction, thus hampering tumor progression. Remarkably, both standard therapies and targeted therapies induce an anti-tumor immune response, while immunotherapies modulate the immune system of cancer patients. However, the success of these therapies relies on the immunogenicity of the tumor antigens expressed by transformed cells [4]. Hence, those tumors that are strongly immunogenic easily elicit an immune response, while recognition by the immune system is greatly impaired when cancer cells are of low immunogenicity [5]. Interestingly, tumor cells can also develop the immune escape hallmark that takes place either by the capacity of cancer cells of hiding or inhibiting the attack by immune cells. In fact, cancer cells can modulate gene expression and signaling pathways of the immune system, inducing an immune-suppressive phenotype that supports tumor growth and metastasis [6]. In this regard, cancer cells also produce several factors that trigger a persistent inflammation that contributes to suppressing anti-tumor immunity [7]. Indeed, while an acute pro-inflammatory response can exert an anti-tumoral effect, chronic inflammation seems to be always detrimental in the cancer context [8]. A proper anti-tumor immune response relies on the optimal function of both the innate and adaptive immunities [9]. Conforming to this, a balance between a pro-inflammatory response, mediated by eicosanoids and cytokines such as IL-1β, tumor necrosis factor-α (TNF-α) and IL-6, and an anti-inflammatory response mediated by pro-resolving mediators, is also crucial [10].

In order to activate the adaptive immune response, the activity of the antigen-presenting cells (APCs), which are represented by macrophages, dendritic cells (DCs) and B cells, is crucial [11]. In this sense, the T cell receptor (TCR) expressed on naïve T lymphocytes must recognize MHC class II molecules loaded with tumoral peptides on the cell surface of APCs. Besides this, the interaction between the CD28 molecule expressed on T cells and the B7 molecule expressed on APCs, represent a costimulatory signal that is essential for the activation of naïve T cells. Upon these interactions, naïve T cells clonally expand and differentiate into effector or memory cells. Effector T cells are subdivided into (i) three types of CD4+ T helper cells (TH1, TH2 and TH17) with different cytokine expression profiles, (ii) regulatory T cells (Tregs) and (iii) cytotoxic T lymphocytes (CTLs) [12]. TH1 cells produce interferon-gamma (IFN-γ) and are necessary for an effective response against intracellular infectious agents and cancer cells. Indeed, TH1 cells are essential for CTLs activation, therefore for the destruction of infected or cancerous cells [13]. For this purpose, TH1 cells produce cytokines that activate the expression of death receptors on cancer cells [14]. On the other side, when TH1 cells are activated erroneously by self-antigens, they are responsible for the development of autoimmune diseases [15]. TH2 cells express IL-4, IL-5, IL-10 and IL-13, which are related to the humoral response [16]. They are involved in immune reactions against allergens and parasitic infections, while, in the cancer context, TH2 cells can favor tumor growth by enhancing angiogenesis and by inhibiting tumor cell killing [17,18]. TH17 cells are characterized by the production of IL-17 and their pro-inflammatory profile that is implicated in autoimmune and inflammatory disorders [19]. The role of TH17 cells in the cancer context is controversial, since some reports indicate a suppressive role on anti-tumor immunity, while other reports describe their role in enhancing recognition of cancer cells [20,21]. However, there is the possibility for TH17 cells recruited to the tumor site to differentiate into the highly immunosuppressive Tregs [22]. Tregs play an outstanding role in maintaining self-tolerance, thus in avoiding autoimmunity and allergies [23,24]. They are characterized by a CD4^+^CD25^high^ phenotype and express the transcriptional factor forkhead box P3 (FOXP3). Tregs favor cancer immune evasion by producing the immune-suppressive cytokines IL-10 and IL-35 and transforming growth factor-β (TGF-β) [25], which hamper APC functions or induce apoptosis of APCs [26]. Finally, CTLs are involved in the elimination of virus-infected or neoplastic cells and exert their cytotoxic activity through the Fas receptor pathway or the release of perforins. The cytotoxicity of CTLs can also be indirect through the release of cytokines such as IFN-γ and TNF-α [27].

Different types of APCs drive differentiation toward a specific type of effector T cell. Macrophages are released from the bone marrow as immature monocytes and, after circulating in the blood vessels, migrate to the target tissues to undergo final differentiation into M1 or M2 mature macrophages. M1 macrophages produce pro-inflammatory factors such as IL-12 and TNF-α and promote TH1 response, with potent microbicidal activity [28]. On the other side, M2 macrophages control inflammation, remove apoptotic cells and sustain tissue repair and are induced by IL-4. M2 macrophages enhance immune suppression by producing the cytokines, IL-10 and TGF-β [29,30], and expressing arginase-1 (Arg-1) [31]. Moreover, they also stimulate tumor growth and progression by producing the vascular endothelial growth factor (VEGF), thus supporting the angiogenesis process [32,33,34].

When the TME is enriched in IL-6, IL-1β and TNF-α, DCs achieve a maturation state that activates the adaptive immune system in order to destroy infected or cancer cells [35]. On the other side, two types of DCs are involved in the maintenance of self-tolerance in physiological conditions, the tolerogenic DCs (tDCs) and immature DCs (iDCs). Both hamper the immune response by expressing low levels of co-stimulatory molecules and producing IL-10 and TGF-β [36,37]. Remarkably, cancer cells enrich the environment with immune suppressive factors and cytokines, such as VEGF, IL-10 and TGF-β, which can either induce tDCs or hamper the maturation of DCs [38,39,40]. Immature DCs (iDCs) are found in peripheral tissues where they recognize and phagocyte pathogens or antigens and reduce the production of pro-inflammatory cytokines. When T cells are stimulated either by iDCs or tDCs, a Treg phenotype or an anergic state are triggered [40,41]. Anergic lymphocytes do not respond to their specific antigens; hence, their effector functions and proliferation are inhibited, resulting in immunological tolerance [42]. B cells are also important for the anti-cancer immune response, because, beside producing IgGs that stimulate the T cell response, they can also directly kill cancer cells [43,44]. However, B cells can also acquire a regulatory phenotype (Bregs) that negatively regulates the immune response by producing IL-10, IL-35 and TGF-β [45].

Another class of immune cells that have been implicated in the negative regulation of the immune system is represented by the myeloid-derived suppressor cells (MDSCs). MDSCs are a heterogeneous population of immature myeloid cells that are important for keeping inflammation and autoimmunity at bay [46,47]. Nevertheless, MDSCs contribute to cancer immune escape by producing reactive oxygen species (ROS) [48] that promote the function of Tregs, leading to a decreased activity of effector cells [49]. Notwithstanding, for eliciting an efficient anti-tumor immunity, a cellular component of the innate immune system, the natural killer cells (NK), deserves special attention. Thanks to the expression of perforins, NK cells permeabilize infected or cancer cells and release granzymes, thus exerting cytotoxic activity [50,51]. The absence of MHC class I molecules that characterize certain cancers represents an activation signal for NK cells [52]. Once activated in this manner, NK cells exert their cytotoxic activity on cancer cells. The absence of MHC class I molecules in certain cancer cells represents an activation signal for NK cells. Once activated in this manner, NK cells exert their cytotoxic activity. In addition, NK cells can kill cancer cells by antibody-dependent cellular cytotoxicity. In this case, the receptor CD16 of NK cells binds to tumor-specific antibodies enabling the recognition of membrane-associated tumoral antigens and ultimately leading to the destruction of cancer cells [53].

In order to mount a proper anti-tumor immune response, it is essential that APCs, T, B and NK cells function properly. However, cancer cells take advantage of several tolerance mechanisms modulating the phenotype of tumor-surrounding cells to create an immune-suppressive TME [54,55]. Understanding the mechanisms and the molecules involved in this microenvironment, including immune cells, is essential in controlling the evolution of the tumor and its impact on the outcomes of patients.

## 3. The Immunosuppressive Properties of the TME

**Figure 1:**
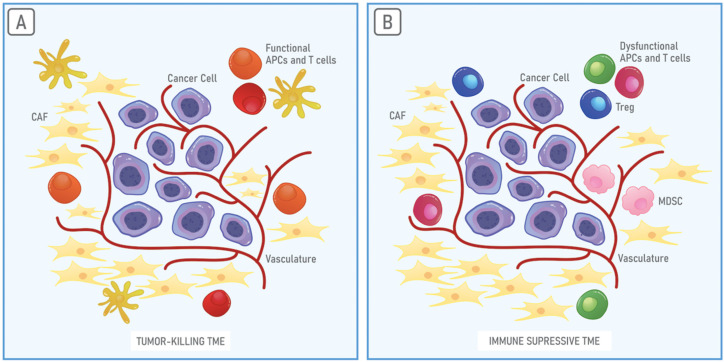
The tumor microenvironment (TME). Cancer cells are surrounded by a complex and heterogeneous environment composed of cancer-associated fibroblasts (CAFs), blood vessel cells and immune system cells, the TME. (**A**) Whether enriched in functional antigen-presenting cells (APCs) or T cells, the TME can promote the recognition of cancer cells by the immune system. (**B**) On the contrary, the TME can be enriched in dysfunctional APCs and T cells, myeloid-derived suppressor cells (MDSCs) and Tregs, creating a highly immune suppressive environment that favors tumor growth and progression.

The tumor and the surrounding environment are in a constant relationship in order to establish and maintain the TME. The TME is a dynamic environment that strongly influences cancer cells and is essential for tumor progression, metastasis and resistance to therapies [56,57]. The TME is characterized by metabolic reprogramming, angiogenesis and dysfunction of the immune system that enables the immune escape [58,59,60]. The TME comprises extracellular matrix (ECM) components, signaling molecules, endothelial cells, cancer-associated fibroblasts (CAFs), immune cells and inflammatory cells [61]. Cancer cells control the functions of the cellular and non-cellular components of the TME through the establishment of signaling networks by releasing soluble factors or extracellular vesicles (EVs), or by cell–cell interactions [62,63]. ECM is composed of water and macromolecules, including glycoproteins, collagens and enzymes that, in physiological conditions, guarantee structural and biochemical support. Collagen is the main component of ECM, functioning as a physical barrier to prevent cell migration; thus, it is responsible to maintain the tissue morphology and organization [64,65]. Hence, in the TME, ECM influences cell adhesion, cell proliferation and cell communication [2]. Metalloproteinases released by cancer cells in the TME assist in the degradation of ECM barriers and allow the release of growth factors that can, in this way, act on endothelial cells, stimulating angiogenesis. Angiogenesis is essential to supply cancer cells with nutrients and supports tumor development and progression [66]. The TME is enriched in CAFs that support the migration of cancer cells from the primary tumor into the bloodstream to colonize distal tissue and organs for metastasis establishment [67,68]. Immune cells, in turn, are the second most abundant in the TME and are prevalently represented by granulocytes, lymphocytes and macrophages [69]. Notably, the immune system of the TME has attracted much attention in the last years to better understand one of the most crucial hallmarks of cancer [1]. The TME can either promote tumor killing by the immune system (Figure 1A) or immune evasion and tumor progression. An immune suppressive TME is characterized by the presence of not functional T cells and APCs that lead to a lack of recognition of tumor antigens. In this case, anti-tumor immunity is further hampered by the presence of several immune suppressive cells, such as Tregs and MDSCs [1,70,71,72] (Figure 1B).

The abundance of tDCs [73,74] and the constant communication between DCs and Tregs are important features of the TME. When contacted by Tregs, DCs suffer changes in their cytoskeleton and enter a lethargic state that impedes T cell priming [75]. Activation of T cells is additionally compromised by the downregulation of co-stimulatory molecules on DCs mediated by Tregs [76]. Moreover, Tregs express the immune checkpoint molecules, CTLA4 and PD-L1, that hamper DCs activity by inhibiting B7. While these molecules are essential to maintain immune response homeostasis, in the cancer context, they prevent the activation of a T-cell mediated anti-cancer immune response [77].

**Figure 2:**
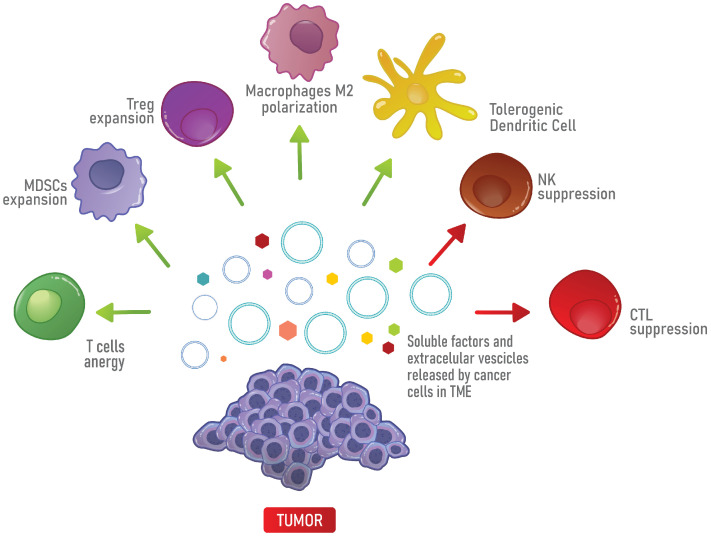
Immune suppression in the TME. In the TME, soluble factors and extracellular vesicles (EVs) released by cancer cells promote the differentiation into immune suppressive cells, such as Tregs, MDSCs, M2 macrophages and tolerogenic DCs, which in turn, inhibit the anti-tumor immune response mediated by T cells, CTLs and NK cells.

Macrophages also contribute to the immunological status of the TME. Monocytes are recruited to the TME and differentiate into tumor-associated macrophages (TAMs), a particular cell type that expresses TGF-β, iNOS and Arg-1 [31,78] and resembles the M2 phenotype [79,80,81]. Interestingly, by secreting TGF-β, TAMs can impair NK cell functions [78]. Moreover, through the secretion of IL-6, TGF-β cancer cells promote the accumulation of MDSCs [82,83], that, in turn, promote the expansion of Tregs [84]. MDSCs also upregulate iNOS and Arg-1 and, in this way, suppress T cell-mediated anti-tumor immunity [73]. Finally, the TME is also characterized by a deficiency in the activity of NK cells and by the recruitment of TH17 that, in turn, attract MDSCs through the production of IL-17 [85]. In addition, TGF-β present in the TME can induce TH17 to differentiate into Tregs [22,86,87] (Figure 2).

In summary, the TME is a complex and specialized environment that is finely tuned by cancer cells and has a great impact on patients’ prognoses. A highly immune suppressive TME is characterized by the presence of tDCs, M2 macrophages, MDSCs and Tregs that avoid the mounting of a proper anti-tumor T cell response. However, other molecules are actively involved, in an intricate pro-tumor signaling network. A protein that has attracted much attention, for its immunomodulatory functions in cancer, is the phospholipid and calcium-binding protein, Annexin A1 [88]. Different studies have been dedicated to discovering its intricate functioning to better establish phenotypic patterns and assist in the design of new therapeutic strategies.

## 4. Structure and Functions of Annexin A1

**Figure 3:**
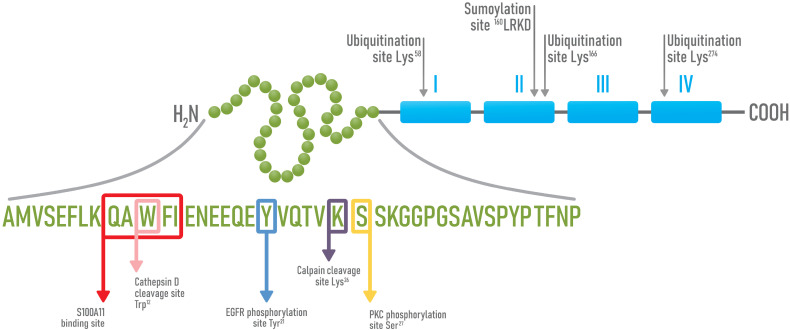
Schematic representation of AnxA1 structure and its sites of post-translational modifications. AnxA1 is composed of an *N*-terminal peptide (green) and a *C*-terminal region, composed of four repetitive domains of about 70 aa each (blue). In the *N*-terminal domain, AnxA1 display sites for EGFR (Y, Tyr21) and PKC dependent phosphorylation (S, Ser27), sites for Cathepsin D (W, Trp12) and Calpain I dependent cleavages (K, Lys26) and a peptide motif (QAWFI), responsible for the binding to S100A11. In the *C*-terminal portion, three sites of ubiquitination (Lys58, Lys166 and Lys276) and a site of sumoylation (160 LRKD) are present.

Discovered in the 1980s, Annexin A1 (AnxA1) is a member of the Annexins superfamily, a group of proteins that share high homology and are characterized by the ability of binding phospholipids in a calcium-dependent manner [89,90,91,92]. AnxA1 is an anti-inflammatory protein that acts on innate immune cell response and is one of the downstream mediators of glucocorticoids [93,94,95]. Besides being highly expressed in neutrophils, monocytes and macrophages [96], AnxA1 is also expressed in several tissues and organs, including the heart, liver, spleen, colon, pancreas, brain, adipose tissue, prostatic tissue and arteries [97,98]. AnxA1 is composed of 346 amino acids (aa) and displays a molecular weight of 37 kDa [99,100]. Structurally, it is composed of a *C*-terminal portion (from Pro44 to Gly344) and an *N*-terminal region (from Ala2 to Asp43) that is specific to AnxA1 [101]. The *C*-terminal portion is highly conserved among Annexins and contains four domains of approximately 70 aa in length each, which are rearranged in 5 helixes responsible for the binding to calcium. These four domains are organized in a compact configuration that is maintained stable by hydrophobic interactions with resistance to proteolysis [102,103,104,105] (Figure 3). Domains 2 and 3 guarantee the binding to calcium and, in this way, mediate the association to membrane phospholipids, while the *N*-terminal region stays away from the membrane and is susceptible to specific interactions with cellular components [106,107]. On the contrary, the *N*-terminal region is extremely variable in length and sequence and confers biological specific activities to the different members of the family [108,109]. A crystallographic analysis showed that the *N*-terminal domain organizes in two alpha helixes (from Ala^2^ to Asn^16^ and from Glu^18^ to Lys^26^) that spatially arrange with a 60° inclination [81]. In the absence of Ca^2+^ ions, the *N*-terminal domain stays close to domain 3, while, in the presence of Ca^2+^ ions, AnxA1 suffers a conformational change that enables its binding to phospholipids through the *C*-terminal region and the exposition of the *N*-terminal domain [101,102,104]. This conformational change allows the amphipathic helix of the *N*-terminal domain to interact with a second phospholipid site that promotes membrane aggregation or allows the interaction with cellular mediators [101].

About the cellular localization, previous studies described AnxA1 expression in the nucleus, cytoplasm and cell membrane [110,111,112,113], which justifies its participation in different processes, such as proliferation, apoptosis, survival, differentiation and migration [109,114]. Regarding the nuclear localization, AnxA1 displays DNA and/or RNA binding sequences and structural motifs and can bind either to double-stranded or single-stranded DNA, in an Mg^2+^ and Ca^2+^ dependent manner, respectively. Furthermore, the helicase activity of AnxA1 has been reported [105,115,116]. In the presence of ATP and Mg^2+^, nuclear AnxA1 binds to DNA and unwinds the double-strand by hydrolysis of ATP. In the presence of Ca^2+^ ions, nuclear AnxA1 binds to two single-stranded DNA molecules, mediating their annealing [115].

Cytoplasmic AnxA1 is distributed throughout the entire intracellular environment, including the inner face of the plasma membrane, linked to vesicular structures, such as early and multivesicular endosomes, phagosomes and, under certain circumstances, to the endoplasmic reticulum [113,117]. In the cytoplasm, by inhibiting the phospholipase A_2_ (PLA_2_), AnxA1 impedes the release of arachidonic acid and its pro-inflammatory metabolites derivatives, such as thromboxane A_2_ and prostaglandins E2, I2, D2 and F2α [92,118,119,120,121,122]. By acting on this lipid metabolism pathway, AnxA1 regulates tissue repair and controls the migration of leukocytes through the endothelium [89,123,124]. Furthermore, the action of AnxA1 in decreasing iNOS levels and in inhibiting the synthesis of prostaglandins, by blocking the expression of cyclooxygenase-2 (COX2), has been demonstrated in macrophages [89,125]. To inhibit PLA_2_, AnxA1 needs to interact with a member of the S100 protein family, S100A11, which is a Ca^2+^ binding protein [126]. The biological functions of this family of proteins are related to the processes of endocytosis, exocytosis, inflammation, cell growth, apoptosis and enzymatic activity regulation [127]. Activation of S100A11, through binding to the Ca^2+^ ion, promotes the recognition of different biological targets, among them, the *N*-terminal domain of AnxA1 [126,128,129].

AnxA1 can also be cleaved at the *N*-terminal domain by elastases [117], metalloproteases [130,131], proteinase 3 [132], by the cysteine protease, Calpain I [133], or by the aspartyl protease Cathepsin D [126,134]. Calpain I cleaves AnxA1 at Lys26, generating a *C*-terminal fragment that displays pro-inflammatory effects through the activation of the ERK1/2 pathway in endothelial cells and the accumulation of the intracellular adhesion molecule 1 (ICAM1). ICAM1 expression leads to the immobilization of neutrophils on endothelial cells and, in this way, increases the transendothelial migration capacity of neutrophils [135]. Furthermore, the cleavage at Trp12 by Cathepsin D also contributes to inflammation, since the fragment of AnxA1 generated has no PLA_2_ inhibitory activity [126]. AnxA1 is also translocated to the cell membrane and externalized or secreted into the extracellular fluid to exert its anti-inflammatory properties [136]. The process of externalization of AnxA1 occurs by three basic mechanisms: (i) through ATP A1 binding cassette transport system (ABCA1), (ii) through phosphorylation of AnxA1 at the Ser27 residue and (iii) through the release of polymorphonucleate gelatinase granules during the degranulation process [93,98,137]. Several groups reported that ABCA1 is a mediator of AnxA1 externalization in cytoplasmic lipid structures, such as microvesicles (MVs). Moreover, ABCA1 also contributes to AnxA1 externalization through lipidation and phosphorylation at Ser27 [98,138,139,140]. In pituitary cell lines, this type of phosphorylation is induced by protein kinase C (PKC) and promotes AnxA1 translocation to the cell surface. However, in order to traffic across the cell membrane, AnxA1 needs to be modified by myristoylation [136]. In fact, AnxA1 contains in its aa sequence potential myristoylation sites localized within the following peptides: Gly30–Ala35, Gly59–Ile65, Gly215–Asn222 and Gly320–Gln325 [136]. Finally, AnxA1 externalization can take place through degranulation, a process that occurs along with chemotaxis and adhesion of polymorphonucleate cells to the endothelial cell monolayer [100,141,142]. Since AnxA1 activation occurs in a calcium-dependent manner, all the processes mentioned above are regulated by the concentrations of Ca^2+^ ions [101].

Once externalized, AnxA1 can interact with formylated peptide receptors (FPR1, 2 and 3) that belong to the G protein-coupled receptors family [143,144]. The main receptor responsible for the anti-inflammatory effects of AnxA1 is FPR2, which is expressed in several cell types, including endothelial cells and stromal cells, being more abundantly expressed in leukocytes [145]. Once activated by AnxA1, FPR2 elicits signaling pathways that inhibit leukocytes migration and adhesion, thus leading to the resolution of inflammation [127,145,146]. FPR2 interacts with a variety of ligands, including the *N*-terminal domain of AnxA1 [147,148]. It is noteworthy that recent studies have shown that FPR2 can be found as a monomer or homo/heterodimer (associated with FPR1 or FPR3). In this context, according to the FPR complex formed, FPR2 ligands can activate multiple pathways, which partially explains the multiple functions exerted by AnxA1 [148,149]. FPR1 was also originally identified for its ability to recognize the N-formylated peptides of bacterial origin and, in this way, to activate phagocytic leukocytes chemotaxis towards the site of infection. This receptor is mainly expressed on leukocytes, such as neutrophils, monocytes/macrophages, NKs and DCs, and has specific endogenous agonists, such as AnxA1 [144,150,151,152,153,154].

AnxA1 displays anti-inflammatory activities by activating apoptosis of neutrophils. Indeed, AnxA1 activates apoptosis by increasing the cytosolic calcium flux that leads to dephosphorylation of the death promoter associated with Bcl-2 (Bad) [137]. Furthermore, apoptotic neutrophils release AnxA1 in order to stimulate their phagocytosis through the recruitment of monocytes. Such a process avoids inflammation, thus ensuring the integrity of the adjacent healthy tissue [155]. AnxA1 also acts in resolving inflammation by decreasing the expression of the intracellular adhesion molecule (ICAM) and of the vascular cell adhesion molecule 1 (VCAM1) that results in the detachment of leukocytes from the endothelium and migration inhibition [156]. Finally, AnxA1 is associated with the process of differentiation of bone and muscle tissue and with the activation and differentiation of T cells into T helper cells [157,158]. Indeed, in T cells, the activation of FPR1 by AnxA1 activates ERK and Akt and increases the TCR signaling pathway. Therefore, the activation of downstream transcription factors, such as the nuclear factor of activated T-cells (NFAT), NF-κB and the activating protein-1 (AP-1), is enhanced [159].

Notably, AnxA1 integrates several signaling pathways that are involved in health and disease. AnxA1 exerts important anti-inflammatory and pro-resolution properties by eliciting signaling pathways through FPRs that inhibit adhesion and migration of leukocytes and by directly inhibiting PLA_2_. However, AnxA1 can also be pro-inflammatory, when cleaved by proteases. Due to its pleiotropic behavior, its role in cancer has been extensively studied in the last years and its expression has been frequently correlated with cancer aggressiveness and resistance to therapies [160,161].

### 4.1. AnxA1 in Cancer

Besides AnxA1 being widely described for its anti-inflammatory activities, its role in cancer development and progression stands out [105]. In this context, it has been suggested that AnxA1 plays a role in malignant transformation, activation of oncogenes, inactivation of tumor suppressor genes, induction of proliferation and cellular invasion [162,163,164,165]. However, AnxA1 can act either as an anti-tumor or as a pro-tumoral factor, depending on the tumor origin and stage [166]. High AnxA1 expression levels have been found in breast cancer [167,168], melanomas [169], hepatocellular carcinomas [170], colorectal cancers [171], gliomas [172], lung adenocarcinomas [173,174] and prostate cancer [175], correlating with worse prognosis, lower disease-free survival rates and lower overall survival [170,174,176]. On the contrary, low expression levels of AnxA1 have been observed in squamous head and neck cancer [177] and thyroid cancer [178] and associate with a worse prognosis, poor differentiation, lower overall survival and higher relapse rates [177,178,179].

Cao and collaborators showed that, despite that breast cancers frequently display downregulation of AnxA1, this protein is upregulated in the triple-negative subtype [180]. Furthermore, AnxA1 has been associated with poorly differentiated breast cancers [167], correlating with lower survival rates [181]. In line with these findings, our group has previously demonstrated that AnxA1 is overexpressed in the triple-negative subtype and lymph node metastasis, when compared to the corresponding primary tumors [168]. Moreover, De Graauw and collaborators demonstrated the crucial role of AnxA1 in supporting the epithelial–mesenchymal transition (EMT) event, promoting migration, invasion and metastasis formation in breast cancer. By using the triple-negative cell line, MDA-MB-231, the authors showed that AnxA1 is essential for the TGF-β signaling, resulting in increased phosphorylation of the small mothers against decapentaplegic homolog 2 (Smad2) and EMT activation [182]. Another study also showed that AnxA1 displays pro-angiogenic functions in breast cancer by supporting the activation of the nuclear factor-kappa B (NF-kB) transcriptional factor [183].

In gastric cancer, AnxA1 up-regulation correlates with advanced stages of the disease and peritoneal dissemination [184], while, in nasopharyngeal cancer, AnxA1 has also been described for promoting migration, invasion and metastasis formation, by mediating autophagy suppression [185]. In lung cancer, a high expression of AnxA1 correlated with the proliferation, invasion, migration and appearance of bone metastasis [157,186].

AnxA1 also exerts important pro-tumoral activities by triggering an FPR1-dependent signaling pathway. In glioblastoma multiforme cells, several studies demonstrated that FPR1 activation mediates tumor cells’ chemotaxis, invasion, proliferation and angiogenesis [187,188,189,190,191]. Huang’s and Zhou’s groups showed that AnxA1 or other ligands released by glioblastoma multiforme necrotic cells would mediate FPR1 activation [189,192]. Beside this, Huang and collaborators also described that FPR1 signaling pathway is responsible for the transactivation of the epidermal growth factor receptor (EGFR) that would result in cancer progression [191]. In colon cancer and hepatocellular carcinoma cells, FPR1 elicits signaling pathways related to chronic inflammation that activate ERK, MAPK and the transcriptional factors, NF-kB and the signal transducer and activator of transcription 3 (STAT3) [158,193,194,195,196]. FPR1 expression was also reported in breast cancer [197,198] and it can be activated, in an autocrine manner, by the *N*-terminal peptide of AnxA1, which is secreted by the triple-negative breast cancer cell line, MDA-MB-231. In these cells, AnxA1/FPR1 autocrine signaling promotes proliferation, migration, invasion and metastatic potential [168]. Nevertheless, it is worth noting that different studies suggest that the role played by FPR1 in carcinogenesis is context-specific. In gastric cancer, FPR1 overexpression was associated with disease progression, as well as lower survival rates [199]. However, other results pointed to FPR1 as a tumor suppressor in this same cancer type [200]. Since the role of FPR1 in carcinogenesis is not fully elucidated, it is crucial to carry on new research in order to elucidate its contribution in different tumors.

In addition to AnxA1 expression and signaling pathways, its cellular localization has been demonstrated to be relevant in the cancer context. Indeed, nuclear AnxA1 has been suggested as a negative prognostic factor of oral squamous cell carcinoma and esophageal squamous cell carcinoma, being associated with lower overall survival [112,201]. In gastric cancer, the nuclear expression of AnxA1 is associated with advanced stages of the disease and with peritoneal dissemination [202]. The involvement of nuclear AnxA1 in tumorigenesis seems to be mediated by a monoubiquitinated form of AnxA1 that is able to introduce DNA mutations [105,203,204,205]. In this sense, insertional mutations can be catalyzed by error-prone DNA polymerases, which are recruited by ubiquitinated nuclear proteins [204,206]. Upon treating cells with mutagenic agents, it was possible to observe an increase in the translocation of cytoplasmic AnxA1 to nuclei, which is likely occurring in response to DNA damage [207]. Another study also demonstrated that upon treatment with mutagenic agents, monoubiquitinated AnxA1 increased in nuclei, indicating the relevance of this type of post-translational modifications during DNA damage response [208].

One of the main causes of cancer lethality relies on the capacity of cancer cells to develop multiple drug resistance [209]. In this context, some studies are indicating the role of AnxA1 in inducing resistance of cancer cells toward chemotherapies. The upregulation of AnxA1 has been correlated with the resistance to adriamycin, melphalan and etoposide. In order to confirm this finding, the authors induced the expression of AnxA1 in an AnxA1-negative breast cancer cell line, MCF-7 and showed that such expression results in resistance to several chemotherapeutic agents. In this same study, the inhibition of AnxA1 expression in an ovarian cancer cell line, SKOV-3, led to increased sensitivity to anti-tumoral drugs [161]. On the other side, in chronic myeloid leukemia, resistance toward chemotherapeutic agents has been associated with a lower expression of AnxA1. Indeed, by suppressing AnxA1 expression in K562 cells, the authors showed that the resistance to anti-tumoral drugs was significantly increased [210].

Worthy of mention, AnxA1 can be differentially phosphorylated in different tumors by EGFR or by the hepatocyte growth factor receptor (HGFR) [211,212,213,214]. Intriguingly, the phosphorylation mediated by EGFR has been described for inducing AnxA1 cleavage, keeping PLA_2_ active in squamous carcinomas [126]. Hence, the differential phosphorylation and the possible subsequent cleavage could explain the effect of AnxA1 on the growth, proliferation, metastasis formation and drug resistance of different tumors and stages of progression [211].

Collectively, the information presented in this section emphasizes the essential role of AnxA1 and its signaling pathway through FPRs in the aggressiveness of several cancers. Indeed, its role in tumor growth, angiogenesis, DNA damage response and migration and invasion of cancer cells have been reported. Besides its anti-inflammatory effect, AnxA1 is profoundly involved in the modulation of the immune system and in the promotion of immune suppression, either in physiological or pathological conditions [148]. Indeed, recently, its role in cancer immune escape has been described [215].

### 4.2. Immunosuppressive Functions of AnxA1 in Physiological and Cancer Contexts

In physiological conditions, it has been shown that AnxA1 promotes immune suppression and resolves the inflammatory process. In this context, AnxA1 enhances the differentiation into M2 macrophages and supports the expression of IL-10 by these cells [216]. When liver cells extracted from mice were treated with exogenous AnxA1, the macrophage M1 phenotype was abolished and an increase in IL-10 expression was observed [217]. Accordingly, other studies demonstrated that treatment of macrophages with a recombinant AnxA1 led to inhibition of iNOS expression, which is accompanied by increased IL-10 levels and decreased IL-12 mRNA levels [218]. The increase in IL-10 expression seems to be due to the FPRs downstream signaling activation of ERK mediated by AnxA1 [192] and may be responsible for the decreased expression of IL-12 in macrophages [218]. Moreover, it has been demonstrated that the tolerogenic nature of cells undergoing apoptosis depends on AnxA1 translocation on the cell surface of these cells. Weyd and collaborators co-cultured apoptotic primary human thymocyte, T cell and neutrophils with DC cells. They showed that AnxA1 expressed on the cell surface of apoptotic cells prevented the activation of toll-like receptors (TLRs) expressed on DCs. The resulting DCs displayed a tolerogenic phenotype that, when it came in contact with CTLs, inhibited their activation, producing IFN-γ and TNF-α [219]. On the other hand, it has been reported that the action of AnxA1 on T cells can vary, according to the environmental conditions and to the state of T cell activation. Gold and others pointed that exogenous AnxA1 can suppress T cell proliferation and limits T cell response [220], but when T cells are stimulated with CD3 and CD28, AnxA1 leads to proliferation and activation of T cells and prolonged activation of Akt, ERK, AP1, NFAT and NF-kB [221]. Furthermore, in activated T cells, the absence of AnxA1 reduces the activation of the TCR downstream signaling pathway [222] and promotes the differentiation into TH2 cells, while inhibiting TH1 response [221]. In another study, Kamal and collaborators described that the mimetic peptide of the *N*-terminal domain of AnxA1 (Acetylated-AMVSEFLKQACYIEKQEQEYVQAVK; Ac2-26) inhibits the proliferation and cytokine production of both TH1 and TH2 [223].

The immune-suppressive activities of AnxA1 can also depend on its presence in EVs. Indeed, AnxA1 can either be secreted or released from the cell within MVs [224]. It is well known that EVs contain cargo molecules such as lipids, proteins and nucleic acids and promote intercellular communication either by binding to the cell surface or by fusing with a specific cellular type [225,226]. Indeed, MVs containing AnxA1 of both normal and cancer cells can alter immune cells’ phenotype [227]. Previously, it was demonstrated that neutrophils produce MVs that, at least in part, due to their AnxA1 content, are capable of stimulating TGF-β expression [228,229]. In agreement with this study, another work demonstrated the immunosuppressive function of neutrophils MVs enriched in AnxA1 and TGF-β. Moreover, these MVs interact with NK cells, decreasing the expression of IFN-γ and TNF-α [230], and with macrophages, increasing TGF-β expression [231].

**Figure 4:**
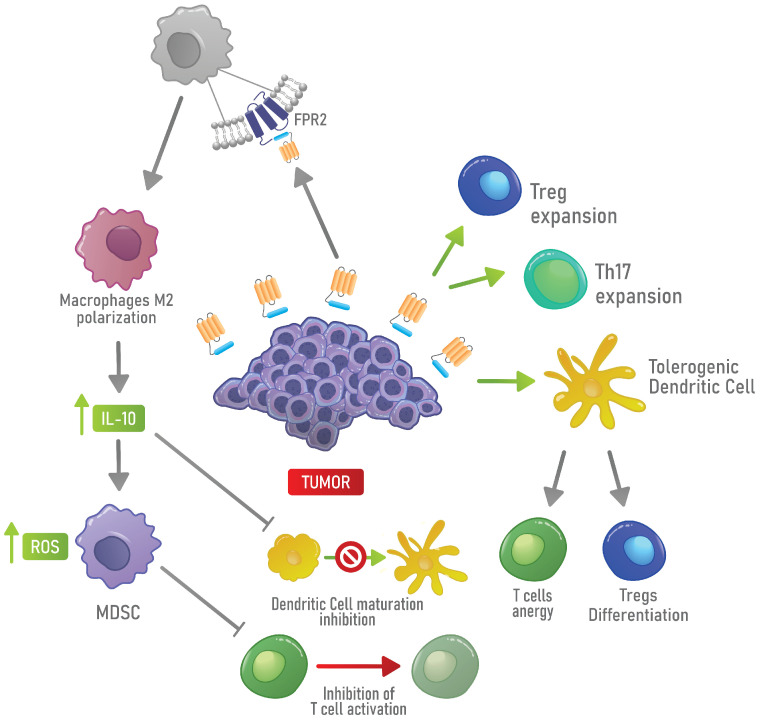
Mechanism of immune escape induced by AnxA1 in the TME. AnxA1 expressed by cancer cells promotes the expansion of Tregs, tDCs and TH17. Moreover, AnxA1 can activate FPR2 on the cell surface of macrophages, supporting the polarization toward an M2 phenotype. In turn, M2 macrophages induce MDSC activity and inhibit the maturation of DCs by producing IL-10.

In the tumor landscape, one of the first indications that AnxA1 could promote immune escape came from experiments in which AnxA1 was knocked down in a glioblastoma multiforme animal model and caused decreased tumor growth and myeloid infiltration. In this work, the authors suggested that AnxA1 acts in highly malignant and actively growing glioblastomas as a chemoattractant for tumor-promoting myeloid cells, especially granulocytes and macrophages [232]. Link and collaborators also described the activity of AnxA1-coated particles in inducing antigen-specific immunosuppression. Indeed, DCs treated with AnxA1-coated particles displayed a tolerogenic phenotype that, in co-culture experiments, promotes an anergic phenotype in T cells, inducing a decrease in proliferation and cytokine secretion [233]. Moraes and collaborators demonstrated, in breast cancer, that, in the absence of AnxA1, macrophages were less sensitive to the polarization toward an M2 phenotype induced by IL-4. Moreover, they showed that macrophages treated with the *N*-terminal peptide of AnxA1 increased the expression of Arg-1, while they decreased the expression of IL-12 [188]. The decrease in IL-12 correlates with cancer progression and metastasis and is probably dependent on the upregulation of IL-10 induced by AnxA1 [215,216,217,218,234]. The role of AnxA1 in polarizing macrophages towards the M2 phenotype in hepatocellular carcinoma has also been described. In this type of cancer, the *N*-terminal peptide of AnxA1 induces a signaling cascade through FPR2 that activates ERK, Akt and NF-kB. Accordingly, a deficiency in FPR2 has been related to a sustained polarization toward an M1 phenotype in hepatocellular carcinoma [216]. Moreover, the role of AnxA1 in inducing TH2 cells infiltration in pancreatic cancer [235] and in inducing the differentiation and expansion of Tregs in the TME of triple-negative breast cancer models has also been described [100]. Bai and collaborators showed that the proliferation of effector T cells, when treated with Ac2-26, was lower and that the function of Tregs was impaired when these cells were treated with the FPR inhibitor Boc1. Boc1 decreased the expression of markers of Tregs, including Granzyme A, IL-2 receptor (CD25) and CCR8. The authors concluded that the AnxA1 signaling regulates the function of Tregs [100]; hence, this could be an ideal target for innovative therapies against breast cancer. A summary of these finding is provided in Figure 4.

**Figure 5:**
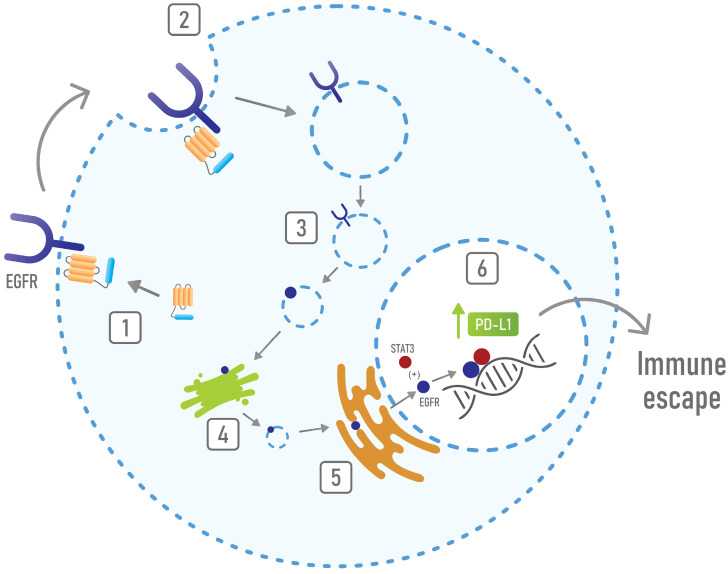
Mechanisms of immune escape mediated by the AnxA1/EGFR interaction. AnxA1 interacts with EGFR (1) and promotes its internalization (2 and 3) and its retrograde transport to nuclei (4 and 5). Once in nuclei, EGFR interacts with STAT3 and induces the expression of PD-L1 (6), thus resulting in immune suppression.

Finally, the activity of AnxA1 in promoting immune suppression in the TME could be related to its interaction with the EGFR [236]. Existing evidence showed that EGFR activation upregulates PD-L1 in lung cancer [237,238], that, in turn, induces the apoptosis of T cells [239]. Confirming this finding, another work showed that the increased expression of PD-L1 is activated by the EGFR through its interaction with STAT3 in nuclei [240]. Moreover, another work described that the EGFR/STAT3 interaction in nuclei is responsible for the expression of iNOS, whose levels correlate with poor overall survival [241]. The role of iNOS expression in cancer cells had been already associated with reduced tumor-infiltrating lymphocytes and with the suppression of anti-tumor immune response [242]. Hence, current evidence suggests that the EGFR/STAT3 interaction induces immune suppression by regulating either PD-L1 or iNOS expression. Indeed, it has been shown that EGFR at the nuclear level functions as a co-transcriptional factor that favors disease progression and resistance to therapies [243]. In the cancer context, it has been shown that AnxA1 promotes the stabilization and the constitutive activation of EGFR [105], as well as the nuclear localization of this receptor [175]. Furthermore, reports showed that, in head and neck cancers, AnxA1 regulates the formation of exosomes and the release of EGFR in these EVs [236,244]. Interestingly, Huang and collaborators demonstrated that, in lung cancer, EGFR-enriched exosomes induce tDCs; tDCs, in turn, induce tumor specific Tregs, which inhibit the specific anti-tumor CTL response. They showed that exosomes containing EGFR are formed thanks to the activity of AnxA1, inducing tumor specific Tregs [236,244]. Based on this, the AnxA1/EGFR interaction seems to play a relevant role in immune suppression in the cancer context. In addition, it has been described that AnxA1 induces STAT3 activation in cancer [245]. Hence, the immune-suppressive properties of AnxA1 also rely on its activities in supporting EGFR signaling and nuclear localization and in promoting STAT3 activation. In this scenario, by inducing the nuclear localization of EGFR, AnxA1 can promote the interaction of this receptor with STAT3 and, subsequently, can induce immune suppression (Figure 5).

Taken together, these findings point towards an important role of AnxA1 in the induction of immune suppression in the cancer context. Therefore, it is possible to draw some future perspectives for cancer treatment that involve the inhibition of AnxA1 signaling. In this scenario, the inhibition of FPRs achieved by Boc1, Boc2, or Cyclosporin H [245] could represent an interesting strategy to induce an anti-tumor immune response. This approach could be used as an isolated therapy, or in association with gold standard immunotherapies, in order to improve the clinical outcome of refractory patients.

## 5. Concluding Remarks

AnxA1 has attracted much attention due to its pleiotropic functions and its involvement in tumor growth and progression. Indeed, its expression and signaling through FPRs are tightly involved in the aggressive behavior of some types of cancer. Recently, the role of AnxA1 in the TME has also emerged and particular effort has been devoted to the description of its immunomodulatory and immune suppressive properties. In this review, we focused on the immune response regulated by AnxA1, which favors M2 macrophages polarization, by increasing the function and proliferation of MDSCs and Tregs and by promoting the enrichment in tDCs and TH17. Therefore, AnxA1 manipulates the TME. Moreover, it is known that AnxA1 regulates the nuclear localization of EGFR and, as a consequence, it can promote immune evasion by favoring the EGFR/STAT3 transcriptional activities in cancer cells. Through these mechanisms, AnxA1 would significantly promote T cell dysfunction and, as a consequence, would enhance immune evasion and tumor progression.

Unraveling the mechanisms by which cancer cells evade the immune system is crucial for the development of new possible therapeutic approaches. Based on the information presented in this review, it is possible to suggest that the inhibition of the AnxA1/FPRs signaling could represent an important and innovative immunotherapy strategy.

## Data Availability

No new data were created or analyzed in this study. Data sharing is not applicable to this article.

## Abbreviations

ABC A1	ATP A1 binding cassette transport system
Ala	alanine
AnxA1	Annexin A1
APC	antigen-presenting cell
Asn	asparagine
Bad	death promoter associated with Bcl-2
Bcl2	B cell lymphoma 2
Bregs	regulatory B lymphocytes
AP-1	activating protein-1
APC	antigen presenting cell
Arg-1	arginase-1
CAF	cancer-associated fibroblast
CCR8	C-C motif chemokine receptor 8
CD	cluster of differentiation
CTL	cytotoxic T lymphocytes
CTLA4	cytotoxic T-lymphocyte antigen 4
DC	dendritic cell
ECM	extracellular matrix
EGFR	epidermal growth factor receptor
EMT	epithelial–mesenchymal transition
ERK	extracellular signal-regulated kinases
EV	extracellular vesicle
FOXP3	factor forkhead box P3
FPR	formylated peptide receptor
Gly	glycine
HGFR	hepatocyte growth factor receptor
ICAM	intracellular adhesion molecule
iDC	immature DC
IFN-γ	interferon-gamma
IL	interleukin Ile, isoleucine
iNOS	inducible nitric oxide synthase
Lys	lysine
MDSC	myeloid-derived suppressor cells
MHC	major histocompatibility complex
MV	microvesicle
NFAT	nuclear factor of activated T-cells
NF-kB	nuclear factor kappa B
NK	natural killer cell
NO	nitric oxide
PD-L1	programmed death-ligand 1
PLA_2_	phospholipase A_2_
ROS	reactive oxygen species
Ser	serine
Smad2	small mothers against decapentaplegic homolog 2
STAT3	signal transducer and activator of transcription 3
TAM	tumor-associated macrophages
TCR	T cell receptor
tDC	tolerogenic DC
TGF-β	transforming growth factor-β
TH	T helper cell
TME	tumor microenvironment
TNF-α	tumor necrosis factor α
Treg	regulatory T cell
Trp	tryptophane
VCAM 1	vascular cell adhesion molecule 1
VEGF	vascular endothelial growth factor
